# Ocular Injuries and Eye Safety Practices in Dental Settings: A Scoping Review

**DOI:** 10.1155/ijod/4079205

**Published:** 2026-10-27

**Authors:** Roma Mascarenhas, Shushma Rao, Kundabala Mala, Shashi U. Rashmi, Prajval Mahajan

**Affiliations:** ^1^ Department of Conservative Dentistry and Endodontics Manipal College of Dental Sciences Mangalore Manipal Academy of Higher Education Manipal India manipal.edu; ^2^ Department of Public Health Dentistry Srinivas Institute of Dental Sciences Mangalore Affiliated to Rajiv Gandhi University of Health Sciences Bangalore Mangalore Karnataka India srinivasgroup.com; ^3^ Department of Conservative Dentistry and Endodontics Manipal College of Dental Sciences Manipal Academy of Higher Education Manipal India manipal.edu; ^4^ Manipal College of Dental Sciences Mangalore Manipal Academy of Higher Education Manipal India manipal.edu

**Keywords:** dentistry, health, ocular injuries, ophthalmology, well-being

## Abstract

**Introduction:**

Aerosols, chemicals, curing lights, dental therapeutic lasers and microbiological pollutants in dental clinics can cause eye infections and impair clinicians, patients and auxiliary vision. This scoping review explored the existing evidence on dental‐related ocular injuries.

**Objective:**

This scoping review aimed to map the evidence on various ocular injuries arising from dental procedures.

**Methods:**

Only published data were searched in PubMed, Scopus, CINAHL (Ebsco) and Embase (Elsevier). English‐language literary data was collected from 2005 to the present. Studies conducted on all patients and dentists, and dental personnel directly involved in dental treatments, working at dental clinics and dental hospitals, were included. The front‐desk employee and non‐direct dental personnel/non‐operating auxiliaries were exempted. Systemic disease‐related and allergic ocular injuries were excluded. Data were imported into Rayyan for critical analysis. Two independent reviewers thoroughly scanned and analysed the selected references for inclusion criteria. A third reviewer was included to resolve article eligibility disputes between the two reviewers. A PRISMA flow diagram and narrative summary were presented to represent the collected data. Data on ocular injuries at the dental set‐up were summarised and explained.

**Results:**

Database search retrieved 49 complete articles. After complete screening, only 8 full articles were included that met the inclusion criteria. The results were obtained from original studies and case reports. Six main themes had emerged from thematic content analysis.

**Conclusions:**

The conclusions of this scoping review will increase awareness among dental clinicians and personnel regarding ocular injuries and support the implementation of appropriate precautionary measures.

**Summary**

• The need for protective eyewear for the clinician and patient during dental treatments. Use of a rubber dam and high‐volume suction should be mandated. Mock drills and safety training for ocular emergencies should be conducted.
• The need for eye or face protection for the clinician and patient during dental treatment when there are chances for exposure to eye or face hazards from flying particles, molten metal, liquid chemicals, acids or caustic liquids, chemical gases or vapours or potentially injurious light radiation.
• Clinicians should wear prescription lenses with a design that can be worn over prescription lenses without disturbing the proper position of the prescription lenses while engaged in operations that involve eye hazards, which provide side protection when there is a hazard from flying objects. Detachable side protectors (e.g. clip‐on or slide‐on side shields) meeting pertinent requirements can be worn.
• The clinician shall use equipment with filter lenses that have a shade number appropriate for the work being performed for protection from injurious light radiation.

## 1. Introduction

Over the years, dentistry has experienced substantial change, characterised by the development of novel methods and innovations [1]. These developments have not, however, removed the possibility of problems, among which can be serious morbidity. Every profession has its own benefits and drawbacks, and maintaining protective well‐being is of supreme significance. Ocular injuries with dentistry are always on the higher‐risk side but can be avoided with proper personal protective equipment [2]. Since the mid‐20th century, observations regarding ocular problems resulting from dental procedures have been made, and in recent years, their incidence has increased [2]. With the COVID‐19 scenario, a lot of emphasis and increased awareness have changed the perception among dentists and patients regarding the need for prompt infection‐control measures and personal safety to prevent cross‐contamination.

Occupational ocular injuries emerging due to mechanical, microbiological and chemical insults result in a dysfunctional ability to work with ease among dental personnel [3, 4]. Occupational ocular injuries in dentistry can emerge from physical factors such as lasers and bleaching/curing lights and electromagnetic irritants causing damage that ranges from mild irritation and corneal abrasions to potentially permanent scleral and retinal damage and cataracts [4]. Literature search has postulated that the dental fraternity is at high risk among the healthcare workers towards ocular injuries [5, 6]. The dentist ought to protect his or her eyes from various chemical and mechanical insults with protective goggles with side shields to prevent lifelong effects on the eyes. Centres for Disease Control and Prevention has postulated that protective glasses should be mandated for both the patients and dentists to prevent cross‐contamination [7]. The Occupational Safety and Health Administration (OSHA) regulations mandate the employer to ensure that each affected employee uses appropriate eye or face protection when exposed to eye or face hazards [8].

The eye infections among the dentists were attributed to insufficient protection of the eyes [9]. This scoping review aimed to map the various dental‐related ocular injuries arising from dental procedures. While Lo et al. [2] in their study (2021) concentrated on acute vision loss, our evaluation adopts a more comprehensive approach, covering a greater range of ocular‐related problems. Numerous healthcare workers lack knowledge of areas that go beyond their areas of expertise, according to previous investigations that show a vacuum in formalised multi‐disciplinary training [10].

## 2. Methodology

Since neither humans nor animals were involved in this investigation, ethics approval was neither required nor obtained. The planned scoping review will adhere to Arksey and O’Malley’s framework [11] and was conducted from 2005 to 2025 and updated in June 2026. The research question formulated was, ‘What evidence exists regarding the types and causes of dento‐ocular injuries and the preventive eye safety practices adopted in dental settings?’ The review protocol was registered for this scoping review under Open Science Network (10.17605/OSF.IO/BZR6C), and the findings of this review are furnished conforming to the PRISMA‐ScR checklist [12].

a. Eligibility criteria: This scoping review included published articles only in English addressing the various ocular injuries arising at the dental set‐up between January 2005 and April 2025, assessing the eye‐related injuries encountered by the dental personnel globally and to ensure any protective measures taken to prevent these episodes. However, this review followed the participants, concept and context (PCC) framework to include the eligible studies for this review [13].
  a. Participants: This scoping review included studies involving all the dental professionals (dentists, dental assistants, dental hygienists, dental nurses and other clinical auxiliary staff directly involved in dental procedures) and patients receiving dental treatment were included. Studies about medical literature and non‐clinical personnel (administrative or front‐desk staff) were considered irrelevant for this study.
  b. Concept: Dento‐ocular injuries connected to dental treatments and routine dental care are the focus of this review. Cross‐sectional studies, cohort studies, experimental studies, case reports and case studies involving dental professionals encountering eye infections due to dental procedures were included. Studies detailing dento‐ocular events connected to dental treatments, such as ocular injuries, infections, trauma, foreign‐body exposure, chemical splashes, aerosol‐related eye hazards, light‐induced ocular damage, exposure‐related eye dangers, awareness regarding ocular protection, precautionary measures, wearing eye protection and risk variables. Studies with ocular injuries due to dental matter like remnants of cavitated matter, spicules of calculus, etc., various instruments emanating aerosol, and dental restorative materials are considered. Studies pertaining to biological, chemical, mechanical or light‐induced ocular dangers in dentistry were included.
   Excluded were studies that solely addressed pre‐existing ophthalmic disorders, ocular symptoms of systemic diseases, allergic ocular conditions unrelated to dental therapy or non‐dental causes of ocular harm. Additionally eliminated were editorials, conference abstracts, letters to the editor, grey literature and research without full‐text accessibility.
  c. Context: The review considered research done in dental care settings, where patients may be exposed to ocular risks associated with dental procedures. Studies, case reports and narrative reviews about ocular injuries due to dental‐induced procedures occurring in dental settings like dental clinics, dental hospitals, academic institutions and community dental camps were included. Dental hospitals were considered important environments for dento‐ocular exposure evaluation because they are specialised dental treatment settings with substantial patient turnover rates, aerosol‐producing processes and extensive usage of rotary instruments, curing lights and chemicals. Studies pertaining to ocular injuries arising in non‐dental medical or hospital settings and tertiary healthcare setups were excluded.

Narrative reviews were incorporated to assist in highlighting frequently reported injury categories, precautionary advice, occupational hazards and current expertise gaps due to the scarcity of high‐quality original research and the segmented character of the published work in this field. The inclusion of narrative reviews allowed for a more comprehensive context‐dependent knowledge of the topic and facilitated the recognition of recurrent themes and concepts throughout the literature, which is consistent with the goal of scoping reviews, which is to investigate and map current evidence as opposed to solely assess intervention effectiveness. Additionally, several narrative reviews offered insightful background data on ocular damage processes, infection‐control issues and eye safety techniques that were pertinent to the review’s goals.

a. Databases and search strategy: A methodical search was carried out across four databases, that is PubMed, Scopus, EMBASE and CINAHL (Ebsco), where the last search was performed in April 2025. In June 2026, the search was revised in all four databases (PubMed, Scopus, EMBASE, and CINAHL [Ebsco]); however, no new records were found beyond those found in the initial search conducted in April 2025 to date. The search string involved keywords like “dental facilities”, “dental clinics”, “dental settings”, “corneal injuries”, “eye infections”, “ocular incidents”, “eye prevention”, “eye safety”, “ophthalmic infections”, “dentists”, “dental auxiliaries”, along with Boolean operators ‘OR’ and ‘AND’. The detailed search string and strategy across the databases are provided in Table 1.
b. Selection process: One author (Roma Mascarenhas) used the search approach to perform the first database search, and duplicates were then eliminated. Titles and abstracts were evaluated by two independent reviewers (Roma Mascarenhas and Shushma Rao), classifying each publication as ’Yes’, ’No’ or ’Maybe’. Following the title and abstract screening, the two reviewers independently screened the full texts of the papers they had chosen. At every point, conflicts were settled via discussion and reviewed by a third reviewer (Kundabala Mala), which resulted in agreement. This scoping review comprised the papers that met the eligibility requirements for data collection and critical evaluation.
c. Charting of the data: Data extraction was performed systematically by one investigator (Roma Mascarenhas) using a standardised, pre‐designed charting format applied uniformly across all eligible full‐text articles. To ensure the accuracy and reliability of the extracted data, a second investigator (Shushma Rao) independently verified a random selection of the charted entries. The pre‐designed format was structured to capture the following parameters: author(s), journal details, year of publication, study title, theoretical and conceptual framework, sample size, study design, causative factors of ocular injuries, types of ocular injuries reported, preventive measures advocated and principal conclusions of the study. Any discrepancies identified during the verification process were resolved through discussion and consensus between the two investigators, thereby ensuring the integrity and consistency of the charted data across all included studies.
d. Critical evaluation of articles: The assessors conducted a qualitative evaluation of the material using the Crowe Critical Appraisal Tool (CCAT) [14] to identify the limitations, research gaps, risk factors and outcome related to this scoping review topic. Using eight categories—preliminaries, introduction, design, sampling, data collection, ethical considerations, results and discussion and so on—CCAT allows for the critical evaluation of different research approaches. Each item had a score ranging from 0 to 5, with a maximum possible score of 40. Publications with a CCAT score of 35 or more were classified as high quality, those with values between 25 and 34 as medium quality, and those with scores below 25 as low quality [15]. One investigator (Roma Mascarenhas) carried out the critical evaluation, while another author (Shushma Rao) randomly chose a portion of the papers for re‐evaluation to guarantee correctness.
e. Generation of results: Thematic qualitative analysis of every chosen article was used to synthesise the results of this scoping review. The coding procedure for theme analysis was carried out independently by two authors (Roma Mascarenhas and Shushma Rao), where the relevant portions of the chosen articles related to the research question on various ocular injuries arising from the dental set‐up were identified and primarily coded using Atlas.Ti [16]. Following a thorough discussion, six primary themes emerged from the final eight selected articles that addressed the research question and were further organised into categories and codes for improved readability and comprehension. At this point, another researcher (Kundabala Mala) participated in the discussion to further develop the codes after the two authors had discussed the initial codes, so they arrived at the final codes.

## 3. Results

Following a preliminary search, 78 articles were chosen according to title evaluation; 29 duplicates were removed from the total. The remainder of 49 publications were then screened based on their titles and abstracts, and 32 of them were considered for a thorough full‐text examination. Eight papers were ultimately determined to be appropriate for inclusion in this scoping review pursuant to the requirements for eligibility. The method of selection of articles has been displayed in the form of a PRISMA flow diagram (Figure 1) [17].

**Figure 1:**
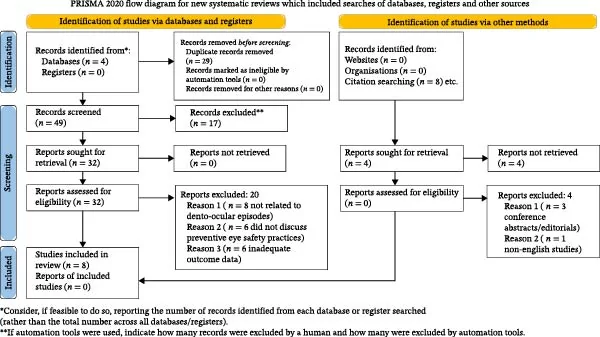
PRISMA flow diagram showing the process of selection of articles. Source: Page MJ, et al. BMJ 2021;372:n71. doi: 10.1136/bmj.n71. This work is licensed under CC BY 4.0. To view a copy of this license, visit https://creativecommons.org/licenses/by/4.0/.

**Table 1:** Detailed search strategy across databases.

Database	Fields chosen	Date accessed	Search strategy	Results popping out	Final
PUBMED	All fields Number 1	7 April 2025	Search: (“dental facilities”[MeSH Terms] OR “dental facilities”[Text Word] OR (“dental clinics”[MeSH Terms] AND “infections”[MeSH Terms]))) AND (“dentists”[MeSH Terms] OR “Dentist”[Text Word] OR (“dental auxiliaries”[MeSH Terms] OR “dental auxiliaries”[Text Word]))	1856	10
	All fields Number 2	7 April 2025	(“corneal injuries”[MeSH Terms] OR “corneal injuries”[Text Word] OR (“eye infections”[MeSH Terms] OR “eye infections”[Text Word]) OR “ocular incidents”[Text Word] OR “eye prevention”[Text Word] OR (“eye injuries”[MeSH Terms] OR “eye injury”[Text Word]) OR (((“Eye”[MeSH Terms] OR “Eye”[All Fields]) AND “safety”[MeSH Terms]) OR “eye safety”[Text Word]) OR (“ophthalmic infections”[Text Word] OR ((“Eye”[MeSH Terms] OR “Eye”[All Fields] OR “ophthalmic”[All Fields] OR “ophthalmically”[All Fields] OR “ophthalmics”[All Fields]) AND “infections”[MeSH Terms]))) AND (“dentists”[MeSH Terms] OR “Dentist”[Text Word] OR (“dental auxiliaries”[MeSH Terms] OR “dental auxiliaries”[Text Word]))	125	0
	All fields Number 3	7 April 2025	(“dental facilities”[MeSH Terms] OR “dental facilities”[Text Word] OR (“dental clinics”[MeSH Terms] OR “dental clinics”[Text Word]) OR “dental settings”[Text Word]) AND (“corneal injuries”[MeSH Terms] OR “corneal injuries”[Text Word] OR (“eye infections”[MeSH Terms] OR “eye infections”[Text Word]) OR “ocular incidents”[Text Word] OR “eye prevention”[Text Word] OR (“eye injuries”[MeSH Terms] OR “eye injury”[Text Word]) OR (((“Eye”[MeSH Terms] OR “Eye”[All Fields]) AND “safety”[MeSH Terms]) OR “eye safety”[Text Word]) OR (“ophthalmic infections”[Text Word] OR ((“Eye”[MeSH Terms] OR “Eye”[All Fields] OR “ophthalmic”[All Fields] OR “ophthalmically”[All Fields] OR “ophthalmics”[All Fields]) AND “infections”[MeSH Terms]))) AND (“dentists”[MeSH Terms] OR “Dentist”[Text Word] OR (“dental auxiliaries”[MeSH Terms] OR “dental auxiliaries”[Text Word]))	15	6
	All fields Number 4	7 April 2025	(“dental facilities”[MeSH Terms] OR “dental facilities”[Text Word] OR (“dental clinics”[MeSH Terms] OR “dental clinics”[Text Word]) OR “dental settings”[Text Word]) AND (“corneal injuries”[MeSH Terms] OR “corneal injuries”[Text Word] OR (“eye infections”[MeSH Terms] OR “eye infections”[Text Word]) OR “ocular incidents”[Text Word] OR “eye prevention”[Text Word] OR (“eye injuries”[MeSH Terms] OR “eye injury”[Text Word]) OR (((“Eye”[MeSH Terms] OR “Eye”[All Fields]) AND “safety”[MeSH Terms]) OR “eye safety”[Text Word]) OR (“ophthalmic infections”[Text Word] OR ((“Eye”[MeSH Terms] OR “Eye”[All Fields] OR “ophthalmic”[All Fields] OR “ophthalmically”[All Fields] OR “ophthalmics”[All Fields]) AND “infections”[MeSH Terms]))) AND (“dentists”[MeSH Terms] OR “Dentist”[Text Word] OR (“dental auxiliaries”[MeSH Terms] OR “dental auxiliaries”[Text Word]))	9	9
	All fields Number 5	6 June 2026	((“dental facilities”[MeSH Terms] OR “dental facilities”[Text Word] OR (“dental clinics”[MeSH Terms] OR “dental clinics”[Text Word]) OR “dental settings”[Text Word]) AND (“corneal injuries”[MeSH Terms] OR “corneal injuries”[Text Word] OR (“eye infections”[MeSH Terms] OR “eye infections”[Text Word]) OR “ocular incidents”[Text Word] OR “eye prevention”[Text Word] OR (“eye injuries”[MeSH Terms] OR “eye injury”[Text Word]) OR (((“Eye”[MeSH Terms] OR “Eye”[All Fields]) AND “safety”[MeSH Terms]) OR “eye safety”[Text Word]) OR (“ophthalmic infections”[Text Word] OR ((“Eye”[MeSH Terms] OR “Eye”[All Fields] OR “ophthalmic”[All Fields] OR “ophthalmically”[All Fields] OR “ophthalmics”[All Fields]) AND “infections”[MeSH Terms]))) AND (“dentists”[MeSH Terms] OR “Dentist”[Text Word] OR (“dental auxiliaries”[MeSH Terms] OR “dental auxiliaries”[Text Word]))) AND (y_1[Filter])	0	0
SCOPUS	All fields Number 1	8 April 2025	((INDEXTERMS(“dental facilities”) OR TITLE‐ABS‐KEY(“dental facilities”) OR (INDEXTERMS(“dental clinics”) OR TITLE‐ABS‐KEY(“dental clinics”)) OR TITLE‐ABS‐KEY(“dental settings”)) AND (INDEXTERMS(“corneal injuries”) OR TITLE‐ABS‐KEY(“corneal injuries”) OR (INDEXTERMS(“eye infections”) OR TITLE‐ABS‐KEY(“eye infections”)) OR TITLE‐ABS‐KEY(“ocular incidents”) OR TITLE‐ABS‐KEY(“eye prevention”) OR (INDEXTERMS(“eye injuries”) OR TITLE‐ABS‐KEY(“eye injury”)) OR (((INDEXTERMS(Eye) OR ALL(Eye)) AND INDEXTERMS(safety)) OR TITLE‐ABS‐KEY(“eye safety”)) OR (TITLE‐ABS‐KEY(“ophthalmic infections”) OR ((INDEXTERMS(Eye) OR ALL(Eye) OR ALL(ophthalmic) OR ALL(ophthalmically) OR ALL(ophthalmics)) AND INDEXTERMS(infectionsauxiliaries”))))/br	20	8
	All fields Number 2	9 April 2025	(INDEXTERMS(“dental facilities”) OR TITLE‐ABS‐KEY(“dental facilities”) OR (INDEXTERMS(“dental clinics”) OR TITLE‐ABS‐KEY(“dental clinics”)) OR TITLE‐ABS‐KEY(“dental settings”)) AND (INDEXTERMS(“corneal injuries”) OR TITLE‐ABS‐KEY(“corneal injuries”) OR (INDEXTERMS(“eye infections”) OR TITLE‐ABS‐KEY(“eye infections”)) OR TITLE‐ABS‐KEY(“ocular incidents”) OR TITLE‐ABS‐KEY(“eye prevention”) OR (INDEXTERMS(“eye injuries”) OR TITLE‐ABS‐KEY(“eye injury”)) OR (((INDEXTERMS(Eye) OR ALL(Eye)) AND INDEXTERMS(safety)) OR TITLE‐ABS‐KEY(“eye safety”)) OR (TITLE‐ABS‐KEY(“ophthalmic infections”) OR ((INDEXTERMS(Eye) OR ALL(Eye) OR ALL(ophthalmic) OR ALL(ophthalmically) OR ALL(ophthalmics)) AND INDEXTERMS(infections)))) AND (INDEXTERMS(dentists) OR TITLE‐ABS‐KEY(Dentist) OR (INDEXTERMS(“dental auxiliaries”) OR TITLE‐ABS‐KEY(“dental auxiliaries”)))	31	31
	All fields Number 3	6 June 2026	((“dental facilities” OR “dental facilities” OR (“dental clinics” OR “dental clinics”) OR “dental settings”) AND (“corneal injuries” OR “corneal injuries” OR (“eye infections” OR “eye infections”) OR “ocular incidents” OR “eye prevention” OR (“eye injuries” OR “eye injury”) OR (((Eye OR Eye) AND safety) OR “eye safety”) OR (“ophthalmic infections” OR ((Eye OR Eye OR ophthalmic OR ophthalmically OR ophthalmics) AND infections))) AND (dentists OR Dentist OR (“dental auxiliaries” OR “dental auxiliaries”))) AND (y_1[Filter])	0	0
EMBASE	All fields Number 1	9 April 2025	(‘dental facilities’/exp OR ‘dental facilities’ OR (‘dental clinics’/exp OR ‘dental clinics’) OR ‘dental settings’) AND (‘corneal injuries’/exp OR ‘corneal injuries’ OR (‘eye infections’/exp OR ‘eye infections’) OR ‘ocular incidents’ OR ‘eye prevention’ OR (‘eye injuries’/exp OR ‘eye injury’) OR (((Eye/exp OR Eye) AND safety/exp) OR ‘eye safety’) OR (‘ophthalmic infections’ OR ((Eye/exp OR Eye OR ophthalmic OR ophthalmically OR ophthalmics) AND infections/exp))) AND (dentists/exp OR Dentist OR (‘dental auxiliaries’/exp OR ‘dental auxiliaries’))	23	23
	All fields Number 2	7 June 2026	((‘dental facilities’/exp OR ‘dental facilities’ OR (‘dental clinics’/exp OR ‘dental clinics’) OR ‘dental settings’) AND (‘corneal injuries’/exp OR ‘corneal injuries’ OR (‘eye infections’/exp OR ‘eye infections’) OR ‘ocular incidents’ OR ‘eye prevention’ OR (‘eye injuries’/exp OR ‘eye injury’) OR (((Eye/exp OR Eye) AND safety/exp) OR ‘eye safety’) OR (‘ophthalmic infections’ OR ((Eye/exp OR Eye OR ophthalmic OR ophthalmically OR ophthalmics) AND infections/exp))) AND (dentists/exp OR Dentist OR (‘dental auxiliaries’/exp OR ‘dental auxiliaries’))) AND (y_1[Filter])	0	0
EBSCO/CINHAL:	All fields Number 1	11 April 2025	((MH “dental facilities+”) OR “dental facilities” OR ((MH “dental clinics+”) OR “dental clinics”) OR “dental settings”) AND ((MH “corneal injuries+”) OR “corneal injuries” OR ((MH “eye infections+”) OR “eye infections”) OR “ocular incidents” OR “eye prevention” OR ((MH “eye injuries+”) OR “eye injury”) OR ((((MH Eye+) OR Eye) AND (MH safety+)) OR “eye safety”) OR (“ophthalmic infections” OR (((MH Eye+) OR Eye OR ophthalmic OR ophthalmically OR ophthalmics) AND (MH infections+)))) AND ((MH dentists+) OR Dentist OR ((MH “dental auxiliaries+”) OR “dental auxiliaries”))	40	20
	All fields Number 2	8 June 2026	(((MH “dental facilities+”) OR “dental facilities” OR ((MH “dental clinics+”) OR “dental clinics”) OR “dental settings”) AND ((MH “corneal injuries+”) OR “corneal injuries” OR ((MH “eye infections+”) OR “eye infections”) OR “ocular incidents” OR “eye prevention” OR ((MH “eye injuries+”) OR “eye injury”) OR ((((MH Eye+) OR Eye) AND (MH safety+)) OR “eye safety”) OR (“ophthalmic infections” OR (((MH Eye+) OR Eye OR ophthalmic OR ophthalmically OR ophthalmics) AND (MH infections+)))) AND ((MH dentists+) OR Dentist OR ((MH “dental auxiliaries+”) OR “dental auxiliaries”))) AND (y_1[Filter])	0	0

### 3.1. Qualities and Attributes of Evidence Sources

Among the 8 papers scrutinised for this scoping review, 7 cross‐sectional studies about this topic and 1 case report were included. According to the eligibility requirements, which covered articles from 2014 to June 2026, the majority of these works were published in the past 10 years. Table 2 provides a thorough summary of the attributes of the papers chosen for this scoping review as well as the information gleaned from these documents.

**Table 2:** Data charted from the included studies.

Title, author(s), journal and year of publication	Purpose of theoretical and conceptual framework	Same size and design	Causes of ocular injuries	Type of ocular injuries	Preventive measures	Conclusion
Knowledge of ocular infections among the 0.dental practitioners across India: a cross‐sectional survey, Arvind and Roma, BMC Research notes, 2024 [17]	This study reviews the knowledge of dentists with the association of eye infections due to dental contamination.	This questionnaire‐based study consisted of 400 registered dentists and academicians.	Micro‐organisms like bacteria, viruses and fungi protozoal infections. Exposure to blood and body fluids All the dental procedures, like scaling, excavations, etc. are accomplished with rotary handpieces spurs of decay, restorative materials, blood products, etc. The aerosols generated from the rotary handpiece are an assemblage of microbes causing infections and deficient protection to the eyes during the treatment procedure.	Scleral and retinal damage Blepharitis to keratitis	66.7% preferred spectacles for protection, whereas 75.0% agreed On Loupes being the best for eye protection	Addition of proper protocol for prevention and preliminary care of eye infections caused owing to dental contamination should be included in the curricula of dentistry. Furthermore, extra facts need to be made accessible to dentists via studies, continual dental schooling, workshops and symposiums.
Risk of eye infections in dental personnel and the need for its prevention: a case report, Ritvi Arvind et al., Journal of Ophthalmic Inflammation and Infection, 2020 [18]	This case report emphasises the importance of preventive barriers for the dentist, and how special protective gear is required for doing restorative cases that involve dealing with infection.	Case report	Frequently executed restorative and operative procedures like caries excavation, restorations, oral prophylaxis, etc., are performed using high‐power‐driven handpieces. A lot of particulate matter like spicules of caries, calculus, amalgam, blood, etc. Eye infections can arise due to a variety of organisms (bacterial, viral, fungal and helminths) Use of power‐driven handpieces which generate aerosols	Mild swelling of the eyelid to complete blindness. Eyelid swelling The particulate matter is lodged in the cornea or conjunctival sac, which causes irritation and redness. Serious eye injuries like perforation, irritation of the lens, might occur due to the particle getting penetrated into deeper tissues. Retinal damage, or the formation of scars and ulcers which can cause obstruction of vision.	Adopt protective glasses and visors as a safe and careful approach when performing the dental procedures. American Dental Association (ADA) and OSHA have demarcated the use of designated protective eyewear with side shields and the use of face shields while performing dental procedures. OSHA recommended the use of glasses that meet OSHA Standard 1910.133(a) (1) and must meet ANSI Standard (Z87.1) to prevent the frontal entry route of the debris. Use of side shields that meet OSHA Standard 1910.133(a) for the prevention of debris travelling sideways. Implementation of an eye wash station within 7.62 m in the vicinity	Ocular injuries can be minimised by the application of standard guidelines. This further spot lights the importance of additional awareness and implementation of protective protection equipment such as a face shield along with protective eye wear.
Occupational ocular incidents in dentists: a multi‐centre study in southwestern Saudi Arabia, Nasser A. Alsabaani et al., International Dental Journal, 2017 [19]	This multi‐centre cross‐sectional study was conducted among dental practitioners working in private, military and Ministry of Health dental clinics in southwestern Saudi Arabia. A pre‐designed self‐report questionnaire was used to elicit information on demographics, splash or foreign‐body ocular incidents, post‐incident practices and use of eye protection.	233 dental practitioners	Ocular incidents as a result of foreign bodies and fluid splashing. Absence of qualification on ocular incidents. Poor compliance with wearing eye protection. Foreign bodies including tooth particles, lubricating oil and restorative mate rials. Inadequate eye protection. Blood‐borne infections, including hepatitis B virus (HBV), hepatitis C virus (HCV) and human immunodeficiency virus (HIV)	Ocular injuries Eye infections	Practitioners’ personal glasses Rinsed eyes under running water Used a handkerchief to clean the eye Use of eye drops Visited the eye clinics	Awareness about eye safety, safe work practices, and infection‐control measures is therefore considered mandatory. It is recommended that the significance of safe work practice and infection‐control policies be emphasised to dental students in their early years of education. Continuous post‐graduate education and training are considered essential for them to remain adherent to the safety guidelines in dental clinics. In addition, dental clinic administrators should provide and promote the use of eye protection.
Light curing procedures performance, knowledge level and safety awareness among dentists, S.E. Kopperud et al., Journal of Dentistry, 2017 [20]	This study aimed to investigate dentists’ exposure to curing light and to obtain information about the dentists’ knowledge on practical use and technical features of their curing lights as well as their safety awareness.	1313 dental participants	UV and visible radiation may induce biological hazards. Blue light, such as that emitted from curing lights, can cause eye damage.	—	Almost one‐third of the dentists used what is considered inadequate eye protection against blue light. The protection shield is mounted to the curing light. Use of orange goggles	This study revealed considerable variations among Norwegian dentists in the Public Dental Service with respect to performance of light curing of restorations, safety awareness and technical knowledge of the curing light.
Shedding light on a potential hazard Dental light‐curing units, Marie T. Fluent et al., Journal of American Dental Association, 2019 [21].	The authors explain the potential risks of using dental LCUs, identify the agencies that provide guidelines designed to protect all workers from excessive exposure to blue light, discuss the selection of appropriate eye protection and provide clinical tips to ensure eye safety when using LCUs.	Review	Chronic exposure to excessive amounts of blue light can damage the light‐sensing cells (photoreceptors) in the retina. The ocular exposure to blue light may be further enhanced when magnification loupes or operating microscopes are used without appropriate filters	Retinal Injury	Eye protection to reduce the amount of exposure to the additional blue light that is present in the dental office. Recommendations for eye protection that focus on impact resistance and potential infection transmission from microorganisms in sprays and spatters. Use of blue‐light protective filters and blue‐light‐blocking eye protection. Do use appropriate barrier sleeves. Do follow recommendations and ensure that all dental personnel use appropriate light‐blocking eye protection during light‐curing procedures.	This review was able to set guidelines and recommendations to use of OSHA regulated protective eyewear. Dental personnel may use inappropriate eyewear, may misuse the eye protection, or may choose not to use any eye protection during light‐curing procedures. More research is needed on the effects of cumulative exposure to blue light in humans to better define the potential hazard from blue light to dental personnel and patients.
Evaluation of Eye Safety Protocol in the Dental Office, Vijayalaxmi Nimma et al. Indian Journal of Occupational and Environmental Medicine, 2024 [22].	This survey aimed to evaluate eye safety protocols in the dental fraternity	150 dentists	Various chemicals, electromagnetic, mechanical, and microbiological irritants commonly used in the dental operatory can cause eye infections and trauma. Injuries caused by infection and trauma Mechanical trauma due to tissue particles, flying projectiles or materials may fly off in each direction with a higher velocity. Cross‐infection, which is caused by Herpes Simplex Type 1 Virus	Irritation to complete vision loss Herpetic keratitis *Chlamydia trachomatis*	Goggles, face shields and visors Visiting a good eye care professional to prevent various eye diseases	Dentists, patients and dental assistants involved in these dental works should also take necessary precautions. Usually, an ocular injury may range from typical eye irritation to complete blindness. Hence, prevention is better than cure.
Occupational health problems among dentists in the Moldavian region of Romania, Lucia Bârlean et al., Revista Medico‐Chirurgicala, 2014 [23].	The aim of this study was to evaluate the occupational health problems among dentists in the Moldavian Region of Romania.	Questionnaire‐based study was conducted on 152 dentists	Exposures by sharp instruments Contact with allergens and toxic chemicals, Direct contact with blood or other contaminated fluids	Ocular injuries Eye infections	Goggles Face shields	Exposure to numerous biological, chemical, environmental, physical, and psychological risk factors is highly associated with clinical activity in the dental office. The dental staff must be informed and educated in order to recognise, control and prevent the potential occupational hazards in the workplace.
Usage of Protective Eye Wear among the Dental Practitioners of Bengaluru City: A Survey with Review of Literature, Rachna Kaul et al., World Journal of Dentistry,2014 [24].	The aim of this study was to find the awareness regarding the occupational eye hazards among dental practitioners of Bengaluru city by assessing the usage of protective eye wear among them.	A questionnaire‐based survey was conducted among 150 dental practitioners of Bengaluru city.	Aerosols containing saliva, gingival liquid, organic dust particles and rich bacterial flora generated during dental procedures can similarly affect the eyes. Lasers and curing light The overall exhaustion of the dental professional	Acute pain, lacrimation and, often, reddening of the eyeball Perforation of the cornea and a consequent injury to the lens. Eye fatigue manifested as heavy eyelids, burning and stinging under the eyelids and sometimes vision‐related issues. Blood‐shot eyes, frequent blinking, lacrimation and increased sensitivity of the eyeball to touch. Decrease in critical frequency (a) delayed accommodative reflex (a) reduced accommodation width and a shift of refraction toward myopia or hypermetropia. The UV radiation actually leads to various biochemical and morphological changes in the eye lens which result in destruction of its cytoskeletal apparatus and deterioration of its functions. Retinal damage	Protective eyeglasses Switching off the artificial light source Both the patient and the dentist should wear protective eyeglasses that have been designed to filter out the harmful wavelengths from the particular LCU	Need to increase the awareness regarding the benefits of protective eye wear usage among dental practitioners so that these deleterious effects can be avoided.

The chosen papers for evidence were evaluated for quality using the CCAT, and the comprehensive ratings for every paper in every domain are shown in Table 3. Six of the articles in the review were classified as medium quality depending on the CCAT ratings, and two were classified as low quality.

**Table 3:** CCAT (Crowe’s Critical Appraisal) scores of the final included studies.

Author, year of publication	Preliminaries	Introduction	Design	Sampling	Data collection	Ethics	Results	Discussion	Total	Quality of the study
Arvind et al., 2024 [17]	3	4	3	3	4	4	3	3	27	Medium
Arvind et al., 2020 [18]	2	4	2	2	2	4	2	3	21	Low
Alsabaani et al., 2017 [19]	4	4	3	3	4	4	3	4	29	Medium
Kopperud et al., 2017 [20]	4	4	4	3	4	4	4	4	31	Medium
Fluent et al., 2019 [21]	4	4	4	4	4	3	3	3	29	Medium
Nimma et al., 2024 [22]	3	3	4	3	4	3	3	3	26	Medium
Bârlean et al., 2014 [23]	2	3	3	2	3	2	3	3	21	Low
Kaul et al., 2014 [24]	4	4	3	4	4	3	4	3	29	Medium

### 3.2. Results Synthesis

Six major themes emerged from the descriptive thematic analysis of the chosen articles (Table 4), notably as follows:

**Table 4:** Themes, categories and codes arising from qualitative analysis.

Themes	Categories	Codes
1. Types of dento‐ocular injuries reported in dental settings	• Infectious ocular conditions • Mechanical ocular injuries • Light‐induced ocular damage • Vision‐related symptoms	• Conjunctivitis, keratitis, blepharitis, herpetic keratitis, protozoal and fungal eye infections • Foreign body injuries, corneal abrasions, perforation injuries, irritation, lacrimation, redness and eyelid swelling • Retinal damage, photochemical injury, blurred vision and photokeratitis due to curing lights and lasers • Burning sensation, eye fatigue, visual disturbances, temporary vision impairment and increased sensitivity to light

2. Causes and contributing risk factors associated with dento‐ocular injuries reported in dental settings	• Biological causes • Chemical causes • Mechanical causes • Light‐related hazards • Contributing risk factors	• Exposure to saliva, blood, splatter, aerosols, bacteria, viruses, fungi and protozoa generated during dental procedures • Exposure to sodium hypochlorite, EDTA, chlorhexidine, lubricating oils, restorative materials and chemical splashes • High‐speed handpieces, ultrasonic scalers, polishing and trimming procedures, flying debris, tooth particles, calculus and restorative materials • Blue light exposure from curing units, UV radiation, prolonged exposure to dental lasers and light‐curing devices • Inadequate PPE use, poor compliance with eye protection, lack of awareness, insufficient training, deficiencies in curriculum and non‐reporting of incidents

3. Aspects of ocular injuries	• Positives	• Protective recommendation • Reason for the low risk with the education
	• Negatives	• Deficient curriculum • Lack of awareness • Low compliance • Non‐reporting incidents • Risk predictors

4. Prevalence of Injury and protection	• Prevalence of ocular injuries • Prevalence of eyewear • Prevalence of patient eye injury • Prevalence of patient eye protection	—

5. Preventive eye safety strategies are adopted in dental settings	• Personal protective equipment • Procedural preventive measures • Engineering and equipment controls • Education and awareness measures • Patient protection measures	• Protective goggles, eyewear with side shields, visors, face shields, orange protective goggles and curing‐light protective filters. • Rubber dam isolation, high‐volume suction, infection‐control practices, hand hygiene and disinfection protocols. • Mounted curing‐light shields, anti‐glare cones, hand‐held shields and eye wash stations. • Safety training, continuing dental education, inclusion of ocular safety in the dental curriculum and awareness regarding OSHA and ANSI guidelines. • Protective eyewear for patients during aerosol‐generating, restorative, polishing and light‐curing procedures.

1. Types of dento‐ocular injuries reported in dental settings
2. Causes and contributing risk factors associated with dento‐ocular injuries reported in dental settings
3. Aspects of Ocular Injuries
4. Prevalence of Injury and Protection
5. Preventive eye safety strategies adopted in dental settings.

Seven of the eight studies included were cross‐sectional surveys, and one was a case study. The research was carried out in several nations, including Saudi Arabia, Romania, Norway and India. Most of the research examined dental practitioners’ awareness of eye protection requirements, preventive measures implemented in dental settings, and occupational ocular potential hazards.

1. Types of dento‐ocular injuries reported in dental settings: As per the included studies, a broad array of dento‐ocular injuries were reported. They were further categorised into (a) infectious ocular diseases such as conjunctivitis, keratitis, blepharitis and herpetic keratitis [18, 19, 22, 23, 25] and (b) mechanical ocular injuries due to rotary instruments, scaling procedures, restorative material remnants, etc. [19, 26], (c) light‐induced ocular damage due to regular use of curing light and dental lasers [21, 22, 25] and lastly (d) vision‐related symptoms like blurring, lacrimation, etc. [18–24, 27].
2. Causes and contributing risk factors associated with dento‐ocular injuries reported in dental settings: As per the full‐text articles, various aetiological factors in ocular injuries in dental settings were narrowed down, arising from the complex multi‐factorial interplay of biological, chemical, mechanical, light‐related and various contributing risk factors.
  Biological causes, like exposure to bacterial, protozoal and viral pathogens [18–24, 27], through splatter, blood, saliva and aerosols produced during standard dental operations. Particularly, when proper eye protection is not worn, these microbes present a danger of conjunctivitis, keratitis and other ocular illnesses.
  Chemical causes primarily involve accidental exposure to splashes, oil lubricants, etc. [18, 19, 24, 27] which may lead to conjunctivitis, corneal burns and temporary vision abnormalities. Inadequate use of eye protection and improper handling of dental materials were often associated with these injuries.
  Mechanical causes were the category that was most frequently reported. These causes were further coded into (a) high‐speed armamentarium like scaling, airotors, etc. [18–24, 27] produced particulate fragments and pieces of broken teeth or restorative materials that could directly injure eyes; (b) light‐curing units [21, 22] were linked to blurred vision irritation and light‐induced retinal damage, particularly after extended exposure; (c) solid material and aerosol created by processes like trimming and polishing presented serious dangers [18–20, 24, 27] and (d) trauma, lack of protective eye wear and PPE [18–24, 27] further exacerbated the occurrence of ocular injuries.
  Light‐related hazards were reported to be one of the most significant dento‐ocular injuries due to prolonged exposure to dental curing light without filtration and dental lasers. When exposed frequently or without appropriate shielding protective equipment, blue light from light‐curing units (LCUs) and UV radiation from dental instruments can result in photochemically mediated retinal damage [19, 21, 22, 25].
  Other contributing risk factors include inadequate use of personal protective equipment and lack of knowledge related to infection control, eye safety and dento‐oculo injuries [18, 19, 23, 24, 27].
3. Aspects of ocular injuries: All the related articles demonstrated further duality into positive aspects and negative aspects based on knowledge and awareness of the dento‐ocular injuries.
  The positive aspects were further coded based on the protection recommended to prevent these injuries with the use of protective glasses with side shields [18, 19, 23, 24, 27] and the reason for the low risk of these ocular injuries due to the presence of well‐equipped knowledge [18, 19, 23, 27]. The regular use of protective eyewear with side shields was highlighted in trials with greater awareness levels. According to this research, the incidence of ocular injuries was considerably decreased by proper training, clinical experience and adherence to infection‐control procedures. A decreased incidence of ocular injuries was the outcome of proactive risk mitigation behaviours exhibited by knowledgeable dental personnel.
  The negative aspects were further coded based on deficiencies in the curriculum [18, 21–24, 27], lack of awareness [18, 19, 23, 24, 27], low compliance [18, 19, 23, 27], non‐reporting of incidents [18, 19, 27] and risk predictors [21–24, 27]. Ocular safety problems in undergraduate and graduate dental curricula have been found in a number of studies. Poor adherence to eye protection regulations was caused by a lack of knowledge and insufficient training. It was regularly observed that ocular incidents were not reported, which was frequently explained by the injuries’ seeming minority or the absence of official reporting procedures. Additionally, several studies found risk factors that contribute to an elevated probability of ocular damage, including years of clinical practice, procedure type, insufficient PPE use and an elevated patient load.
4. Prevalence of injury and protection: This aspect was further categorised based on (a) prevalence of ocular injuries [18, 19, 21–24, 27], (b) prevalence of eyewear [18–24, 27], (c) prevalence of patient ocular injury [18, 19] and (d) prevalence of patient eye protection [18, 19, 22, 23]. The prevalence statistics showed that dental personnel had a significant burden of ocular injuries, ranging from minor discomfort to serious trauma. In spite of all this, there was an irregular use of protective eyewear. Although there was an overall substantial amount of knowledge of eye safety, actual implementation varied greatly. Even though they are less common, patient‐associated ocular injuries highlight how vulnerable patients are during aerosol‐generating and polishing procedures. Patients’ eye protection was discovered to be inadequate, pointing to a serious weakness in all‐encompassing dental safety procedures.
5. Preventive eye safety strategies adopted in dental settings: Several safety measures implemented in dental settings to reduce the incidence of dento‐ocular events among dental professionals, auxiliary personnel and patients were emphasised in the included research. This was further categorised into (a) personal protective equipment related to safety eyeglasses [18, 19], face shields along with protective eye wear [19, 23], visors [20, 23], protection shields mounted onto the curing light [21, 22], separate hand‐held shields with eye protection [21, 22], orange round or oval shields, orange goggles [22], anti‐glare cones on light guide [22], curved paddles for dentist and patient [22] and goggles [24]. (b) Procedural preventive measures like oral prophylaxis [18, 19], restorative treatment [19], trimming and polishing [20, 23], orthodontic procedures [20], etc. (c) Engineering and equipment controls like the use of eyewash station [28–30], use of chemical neutralisers (carboxymethyl cellulose sodium, tobramycin, etc.) [19], technologies including orange‐tinted protection devices, curved paddles and anti‐glare cones [22]. (d) Education and awareness measures by including instruction on ocular protection and dental programmes for both undergraduate and graduate students [19], instruction on infection prevention procedures and proper PPE use [18, 19], (e) Patient protection measures with the use of rubber dam, high‐volume suction, protective eye wear for the patient during the procedures, etc. [18, 19, 22, 28–30]. A variety of apparatus‐based and personal protective measures were included in the mitigation strategies found in the literature. The most often suggested remedies were safety goggles and spectacles. Substantial risk mitigation was seen when face shields were used in conjunction with eyewear, visors and specialised curative light shields.

## 4. Discussion

This scoping review divulged the various ocular injuries acquired due to dentistry. These infections can occur at any stage of a dental procedure. This scoping review proposed to analyse the existing collected evidence on the ocular injuries arising from the dental set‐up, focusing particularly on determining the research gaps. In addition, the observations from thematic content analysis enlighten us on the future practices that could be included in the day‐to‐day practice of a dental professional to protect the eyes from ocular injuries that arise from the dental practice during various treatments, safety training, and environmental controls, and also by adhering to guidelines from organisations like the OSHA and the American National Standards Institute (ANSI) which help to minimise these occupational risks, thereby improving the quality of clinical work and personal well‐being [23, 24, 27, 28].

### 4.1. Ocular Injuries Arising From Dental Treatments

The eyes are highly sensitive and may be exposed to a wide variety of different dental infections and injuries. The principal risk factor for the spread of infections in dental practice is the production of aerosols by high‐speed rotary instruments, for example by restorative procedures. The high‐speed aerosol can contain a variety of different particles, including those of restorative materials, dental plaque and bur fragments. These particles can be propelled by the air with high velocity, up to 96 km/h, and thus become contact agents for the spread of infections, as suggested by R. von Krammer [28]. Production of aerosols by dry cutting of tooth structures without the use of water as a coolant can also cause an increase in the number of ocular infections [18]. The oil from the handpiece and solid fragments produced by the frictional heat of cutting can be particularly hazardous to the eyes [23, 29]. A rubber dam with high suction and appropriate personal protective equipment are essential in preventing such injuries [19, 31].

The risk of chemical splash in the dental surgery involves both the patient and the dentist. Some of the most common chemicals used in dental practice are caustic in nature and can cause severe damage to the eye [19]. Some of the chemicals used in dental practice are sodium hypochlorite, chlorhexidine gluconate and EDTA. There is a recognised risk of transfer of chemicals from gloved hands to the periocular skin [26]. Salivary bacteria are also capable of transmitting disease, and studies have shown that these bacteria remain capable of growth in the ambient air for up to 30 min. It is well recognised that in the presence of sufficient numbers, these organisms can overcome the natural defence mechanisms of the eye and establish infection [18, 22]. To prevent such problems, provision of an eyewash station [18, 28–30] and use of a chemical neutraliser such as carboxymethyl cellulose sodium (sodium carboxymethylcellulose) and tobramycin, for example, are recommended [19].

### 4.2. Types of Ocular Injuries Arising From Dental Treatments

Ocular injuries caused by dental procedures are rare, and only a few cases have been described in the literature. Their clinical presentation can be of a light, self‐limiting type (lacrimation, foreign‐body sensation, purulent discharge, etc.) but can also be of a more severe type and have serious sequelae [18]. *Chlamydia trachomatis* has been found in cases of conjunctivitis with purulent discharge, which can have serious sequelae of conjunctival scarring and corneal vascularisation [18, 23, 29]. *Bacterial blepharitis* caused by Staphylococcus species has also been reported in the dental environment [19, 31]. A serious type of eye injury is *Herpes keratitis*, which can be caused by contact with saliva of a person infected with Herpes Simplex Virus Type 1 [22, 26]. The prognosis for this type of injury is particularly poor. Use of facial shields, donning of double gloves, disinfection of equipment to reduce the microbial load and hand washing with hot water are recommended [18, 19, 26].

### 4.3. Ocular Hazard Due to Lasers and Dental Curing Lights

The use of high‐intensity lights in dentistry has been seen to pose ocular hazards. Lasers and curing lights cause visual deterioration if proper measures are not instituted. Dental LCUs are strong blue‐light emitters that can harm the eyes and damage soft tissues [22]. Long‐term exposition to UVA and blue light can harm the eyes [22]. The wavelengths at which blue light and UV radiation cause the most ocular damage are 440 and 270 nm, respectively. The retina absorbs blue light once it passes through the ocular medium. Blue light increases retinal ageing and degeneration at long‐term low exposure levels by photochemically harming the retina’s choroid and pigmented epithelium [22, 32]. In contrast to blue light, UV light tends to pass through the conjunctiva, cornea and lens before entering the retina. Therefore, UVA rays promote both cataractogenesis and corneal damage, also known as photokeratitis [19, 21, 25]. Therefore, it is important for both the dentist and the patient to make sure that they are wearing appropriate and efficient eye protection [19]. Using the eye gear recommended by the manufacturer of the LCU is the best course of action [21].

### 4.4. Safety Measures

Dental practitioners should prefer eyewear with side shields that meet OSHA Standards 1910.133(a) to prevent the frontal and sideways entry of debris [18, 27, 28]. OSHA has also regulated the dental healthcare workers to use mouth masks and face shields, which conform to ANSI Z87.1‐1989/ANSI Z87.1‐2003/ANSI/ISEA Z87.1‐2010 Standard [5]. Cleaning and disinfection of the protective eyewear devices should be thoroughly followed according to the manufacturer’s protocols with gloves [4]. Rubber dam and high‐volume evacuation systems should be used regularly to prevent splatter and adhere to strict infection‐control protocols [5, 18, 25]. It is highly recommended that dental clinicians visit an ophthalmologist for regular eye visits to maintain their eye competency [3, 5].

### 4.5. Future Perspective

Given the rising frequency of occupational ocular injuries among dental professionals worldwide, the results of this scoping review highlight the need for more methodologically rigorous research in this field. In order to protect ocular health and improve clinical safety in the dental field, this evaluation promotes the creation and application of official guidelines and policy statements.

### 4.6. Policy Statement: Prevention is the Key

In the dental field, ocular injuries are a major but mostly avoidable occupational risk. The evidence compiled in this review demonstrates that dento‐ocular injuries result from biological, chemical and mechanical causes during routine dental procedures. These injuries are exacerbated by low awareness, inadequate use of personal protective equipment and shortcomings in professional education and incident reporting.

Regardless of the perceived amount of risk, it is advised that all dental professionals, auxiliary workers and patients be required to wear eye protection throughout all clinical procedures. To protect against aerosols, splatter, solid debris, chemical exposure and phototoxic risks from LCUs, protective eyewear must meet established safety standards and have side shields or similar lateral coverage [25].

Ocular safety should be methodically included in dental education curricula by dental schools and regulatory agencies. This should include thorough instruction on risk assessment, preventive measures and proper handling of ocular injuries. Programmes for ongoing professional development should prioritise adherence to eye protection laws and offer information on new technologies and risk‐reduction techniques. All dental service settings should implement standard safety practices, such as routine oral prophylaxis and the requirement to wear safety glasses in addition to face shields or visors during aerosol‐generating operations.

## 5. Limitations and Research Gaps

This evaluation found several significant methodological and practice‐associated flaws in the body of research on dento‐ocular events. The preponderance of cross‐sectional and observational studies and the dearth of longitudinal, standardised, prospective or interventional research were significant research limitations. As a result, little is known about the long‐term effects, causal connections and efficacy of preventive measures.

All the incorporated studies had clear reporting restrictions. At present, there is no standardised reporting or monitoring system for recording dento‐ocular injuries, and ocular events in dental settings seem to be significantly underreported. Comparison and synthesis between studies were further hampered by differences in nomenclature, injury classification and outcome reporting.

Additionally, a few practice‐related gaps were found. Despite extensive knowledge about occupational eye dangers, dental personnel’s adherence to protective eye protection protocols varied among research. In many clinical situations, patient eye protection protocols were documented less frequently and seemed to be implemented insufficiently. These results imply that standard dental procedures do not always incorporate eye safety precautions. Hence, strict enforcement of guidelines pertaining to eye safety in dental clinics should be mandated.

Therefore, well‐constructed interventional investigations, the creation of standardised systems for reporting ocular injuries, and the assessment of methods to enhance patient and clinician compliance with eye protection measures should be the main areas of future study.

## 6. Conclusion

• The results of this scoping review indicate that eye safety issues and ocular injuries are pertinent work‐related risks in dental settings. Among the most frequently reported problems in all of the included trials were mechanical injuries, foreign‐body exposure and insufficient usage of protective eyewear. Nevertheless, the majority of the information that is currently available comes from cross‐sectional surveys, descriptive studies and isolated case reports, which makes it difficult to establish causal linkages or assess the efficacy of certain preventative strategies.
• Even though the reviewed literature suggests that wearing eye protection could help lower ocular hazards in dentistry settings, the available data is still sparse and inconsistent. As a result, the results should be read with caution and seen more as a summary of the available data than as conclusive evidence of efficacy. To gain a better understanding of the prevalence, risk factors and preventative efficacy of eye safety measures in dentistry practice, more carefully planned interventional studies are required.
• Prevention is unquestionably better than reactive treatment when it comes to managing occupational ocular risk. Every dentist has a fundamental professional duty to prioritise health and safety over all other factors when performing clinical procedures.
• It is the professional duty of dental clinicians to make sure that all chairside assistants and other ancillary staff members are well informed about the type and seriousness of occupational ocular dangers that are present in the dental practice. This includes both theoretical understanding and the proper and reliable use of personal protective equipment. To guarantee ongoing compliance in all clinical settings, standardised eye protection procedures must be legally created, strictly enforced and routinely reviewed.

## Author Contributions

**Roma Mascarenhas**: conceptualisation, methodology, investigation, data curation, formal analysis, visualisation, writing – original draft, writing – review and editing, contributed to the development of the review concept and design, participated in data acquisition, analysis and interpretation, prepared the initial draft of the manuscript, and coordinated the overall writing process. **Shushma Rao:** conceptualisation, methodology, investigation, data curation, formal analysis, writing – original draft, writing – review and Editing, contributed to the conceptual framework of the review, participated in literature acquisition and interpretation of data, assisted in manuscript drafting and critically reviewed the intellectual content of the manuscript. **Prajval Mahajan:** investigation, data curation, formal analysis, validation, writing – original draft, writing – review and editing, contributed to data collection, organisation, analysis, interpretation of findings and assisted in drafting and revising the manuscript. **Kundabala Mala**: supervision, validation, writing – review and editing, provided academic supervision, critically reviewed the manuscript for important intellectual content and contributed to the refinement of the final version. **Shashi U. Rashmi**: supervision, validation, writing – review and editing, contributed to the critical revision of the manuscript, provided expert guidance on interpretation and presentation of the review findings and approved the final manuscript for submission.

## Funding

This study did not receive any specific funding.

## Disclosure

All authors have read and approved the final version of the manuscript. Kundabala Mala (corresponding author/manuscript guarantor) had full access to all the data in this study and takes complete responsibility for the integrity of the data and the accuracy of the data analysis.

## Ethics Statement

Ethical clearance was not required for this scoping review, as this study is an evidence synthesis study whereby the analysis and synthesis of data from published literature and publicly available sources are undertaken. Thus, there are no human participants, patient data, confidential data or personal data (identifiable or otherwise) that are involved in this review. In line with guidelines for evidence synthesis studies, therefore, no institutional ethical clearance nor consent is required for this review.

## Conflicts of Interest

The authors declare no conflicts of interest.

## Data Availability

The data that support the findings of this study are available from the corresponding author upon reasonable request.
